# Enterprise service-oriented transformation and sustainable development driven by digital technology

**DOI:** 10.1038/s41598-024-60922-w

**Published:** 2024-05-02

**Authors:** Shuangcheng Luo, Jianjiang Liu

**Affiliations:** 1 School of Economics, Hunan Agricultural University, Changsha, 410128 China; 2 School of Economics and Management, Changsha University of Science and Technology, Changsha, 410076 China

**Keywords:** Sustainable development, Digital technology application, Service-oriented transformation, ESG, Technological innovation, Environmental economics, Sustainability

## Abstract

The deep integration of digital technology and the real economy not only affects the production and operation mode of enterprises, but also becomes the promoter of service-oriented transformation and the driving force of sustainable development. Based on the text analysis method, this paper uses the data of Chinese listed manufacturing enterprises from 2011 to 2020 to study the impact of digital technology application on the service-oriented transformation and sustainable development of enterprises. It is found that digital technology application significantly improves the environmental performance and economic performance of enterprises by driving their service-oriented transformation and technological innovation, and then enhances their sustainable development. The improvement effect of digital technology application on the sustainable development of resource-based enterprises and capital-intensive enterprises is more significant. The conclusion in this paper provides micro-evidence for understanding the role of digital technology in addressing environmental issues and sustainable development.

## Introduction

With industrialization and growing population size, the world is facing many environmental challenges such as climate warming and frequent occurrence of extreme weather^1^. As the world's largest developing country, China is still a major global energy consumer and carbon emitter. The rapid development of industrialization and urbanization makes China's energy consumption rigid and demand remains strong, and its carbon dioxide emissions account for 29% of the world's emissions, exceeding those of the United States and Europe combined, and have not yet reached the peak^2^. In response to environmental and climate challenges, China has endeavored to pursue the strategic goal of sustainable development, and the State Council has proposed in its five-year plan for energy conservation and emission reduction that energy consumption per unit of gross domestic product should be reduced by 13.5% from 2020 to 2025^3^. In September 2020, the leader of the Chinese government proposed at the 75th session of the United Nations General Assembly that China's goal is to achieve CO_2_ emissions to peak by 2030 and to achieve carbon neutrality by 2060 (Dual-carbon target). Under the constraints of the Dual-carbon target, it is crucial for manufacturing companies to reduce their dependence on environmental resources by transforming their production methods. China's experience can also serve as a useful reference for other countries, especially developing countries, on how to achieve sustainable development.

At the same time, with the continuous iteration of the new generation information technology and the rapid growth of the Internet user group, digital technology is widely used in people's production and life, gradually becoming a new engine of economic growth^4^. Data shows that the scale of China's digital economy has increased from 16.1 trillion RMB in 2014 to 50.2 trillion RMB in 2022, with an average annual growth rate of 15.3%, much higher than the GDP growth rate during the same period. The proportion of digital economy in GDP has also increased from 25% to 41.5%, which has a profound impact on the economy and society. Digital technologies such as artificial intelligence, blockchain, and big data are widely used in enterprises, which have a significant impact on information communication, transaction costs, and other aspects of the enterprise's production process, and the enterprises are gradually driving digital transformation^5^. At the policy level, governments of most countries have gradually realized the huge potential of digital transformation, and have issued corresponding policies to support digital development^6^. It can be seen that the application of digital technology has important practical significance for the long-term development of world's economy in the future.

More and more literature is paying attention to the application value of digital technology. On the one hand, it explores the relationship between digitalization and business performance from a micro perspective^7,8^. On the other hand, it analyzes the impact of the digital economy on macroeconomic stability, carbon emissions, and other aspects from a macro perspective^9–11^. The application of digital technology has also had a significant impact on the service-oriented transformation of enterprises, including monitoring, controlling, optimizing production and business management processes, improving customization efficiency, and providing intelligent solutions to customers^12^, becoming the driving force for service-oriented transformation of enterprises^13^. Another related literature explores the important role of service-oriented transformation of enterprises in influencing environmental performance of enterprises^14^. However, not many studies have explored the relationship between digital technology applications and sustainable development from a servitization perspective, and there is a lack of relevant empirical evidence. The degree of environmental dependence varies greatly across industries, with an increase in the share of the service industry reducing pollutant and carbon dioxide emissions^15^, while the manufacturing industry has a higher degree of environmental dependence. For this reason, we take the manufacturing industry as our research object to explore how digital technology application affects enterprise sustainable development? How do digital technology applications and servitization interact, and what are the resulting implications for sustainable development?

The purpose of this paper is to enrich related environmental research and provide useful insights for enterprises to formulate sustainable development strategies by exploring the impact mechanism of digital technology application on enterprise sustainable development from both direct and indirect effects, and the role of service-oriented transformation in it. Moreover, using the data of 607 listed manufacturing companies in China from 2011 to 2020, this paper carries out empirical testing and finds that the application of digital technology not only reduces transaction costs and improves the efficiency of operation and management, but also reduces the waste of resources and environmental pollution by improving the efficiency of resource use, which effectively promotes the sustainable development of companies. This paper provides a new perspective for the sustainable development of manufacturing enterprises. The application of digital technology to provide customers with more efficient, higher quality products and services, to expand higher value-added services for the enterprise, to improve operational performance and improve environmental performance. IBM is a good example^16,17^. In the beginning, it is a computer parts maker, with increasingly stringent environmental regulations, the firm outsources the low value-added manufacturing sector, and focused on services such as R&D, design, brand marketing, and gradually transformed itself into an intelligent service provider, which has resulted in significant improvements in the company's financial and environmental performance.

Compared with the existing literature, the contribution of this paper is reflected in three aspects. Firstly, based on the transaction cost theory and resource dependence theory, it explores the intrinsic mechanism of the impact of the application of digital technology on the sustainable development of enterprises, which further enriches the relevant research in the field of sustainable development. Secondly, it explores the mediating mechanism of the impact of the application of digital technology on the sustainable development of enterprises from the interaction between digital technology application and servitization, which provides a new perspective for the realization of the sustainable development of enterprises. Unlike recently research who explored the facilitating role of digitalization from the perspective of digital green innovation^18^, this paper explores new mechanisms for digital technologies to influence sustainable development from the perspective of servitization. Thirdly, by manually organizing the annual reports of Chinese listed companies and using Python tools to count the word frequencies of keywords related to digital technology application in the annual reports to objectively reflect the degree of digital technology application of enterprises, and to examine the heterogeneity analysis of digital technology application on sustainable development from the aspects of industry attributes and industry clustering characteristics, which expands the research in this field.

The following is the arrangement of this article. The second part is a review of relevant literature. The third part is theoretical analysis, based on which the research hypotheses of this article are proposed. The fourth part is the empirical model and variable selection. The fifth part is the empirical results and analysis of the application of digital technology on sustainable development. The sixth part further empirically analyzes the heterogeneity and mechanism of digital technology application on sustainable development. The seventh part is the conclusion and research prospects.

## Literature review

A literature closely related to this article explores the impact of digitization on circular economy and sustainable development. Digitalization can help businesses develop sustainable circular products^19^ and is an important driving factor for the circular economy^20^. Specifically, the Internet of Things technology monitors products and components throughout the life cycle through the interconnection of sensors and electronic devices with physical devices, providing technical support for sustainable product development, reducing waste and improving resource efficiency^21,22^. Big data analysis controls the industrial production process, and improves the matching efficiency of waste and resources by collecting and processing input and output information in real time^23^. Big data analysis can be also used to predict product quality, reduce production downtime, schedule maintenance, optimize energy consumption, and more^24^. Enterprises' digital transformation has become an important driving force for reducing carbon emission intensity, and improve enterprises' technological innovation, internal control and environmental information disclosure capabilities^25^. Some research pointed out that the application of digital technologies in the financial field can provide enterprises with convenient services and lower the financing threshold, reduce the information asymmetry in the transaction process, improve the financing difficulties of enterprises, and improve the energy and environmental performance by improving the green innovation of enterprises^2^. Digital transformation has a strong role in promoting enterprise economic performance, but its impact on environmental performance shows an inverted U-shaped relationship^26^. Some studies have found from a macro perspective that digital infrastructure construction contributes to the "energy conservation, emission reduction" and sustainable development of regional economic development^27,28^.

Another literature mainly focuses on the field of servitization, and on this basis, advances in exploring the impact of digital service-oriented transformation on sustainable development. Enterprise service-oriented transformation is the process of transforming a company from a product centric approach to a product plus service oriented approach^29^. Manufacturing companies represented by IBM, Rolls-Royce, and others provide services to customers based on products to improve customer satisfaction, reduce operating costs, and gain customer loyalty^30^. Compared to traditional manufacturing models, service-oriented manufacturing models have new characteristics such as integration, value-added, and innovation. More and more manufacturing enterprises are adopting service strategies to promote business growth and create competitive advantages^31,32^. Servitization not only benefits the competitiveness of enterprises, but also improves their environmental performance by increasing the recycling of products. Firstly, servitization services bring potential benefits, such as strengthening customer relationships, creating higher barriers for competitors, and generating new revenue streams^33,34^, which are excellent tools for enhancing competitiveness and promoting sustainability^35^. Secondly, service-oriented transformation can reduce the amount of waste generated at the end of the lifecycle and reduce the consumption of raw materials. Some research explored the environmental performance of rental and refurbishment services provided by stroller companies^36^. Service models such as renting jeans and recycling, and Philips lighting paid services have also proven that servitization can improve the environmental performance of enterprises^14^. Some research used data from 208 manufacturing companies in Europe to study and found that servitization can improve energy efficiency, thereby improving environmental performance of enterprises^37^.

Digital technologies have significant advantages in driving servitization transformation^13,38^. Some research found that there needs to be an effective interplay between digitization and servitization^39^, and that without this interplay manufacturing firms may face the paradox of digitization, whereby increased revenues from digital services do not lead to greater profitability due to sharply increased costs. Digital technology improves quality and increases efficiency, but similarly, the cost of services is pushed up by the increasing availability of more advanced solutions and functionality, which requires higher introductory investment and maintenance costs^12^.

Currently, most of the existing literature has explored the relationship between digital technology and sustainable development as well as servitization and environmental performance. However, there are no studies on the interaction between digital technology, servitization and sustainable development, and the theoretical elaboration of the mechanisms of how digital technology and servicization interactions affect sustainable development is not clear, and there is a lack of corresponding empirical analysis and evidence.

## Theoretical basis and research assumptions

### Theoretical basis

**Figure 1:**
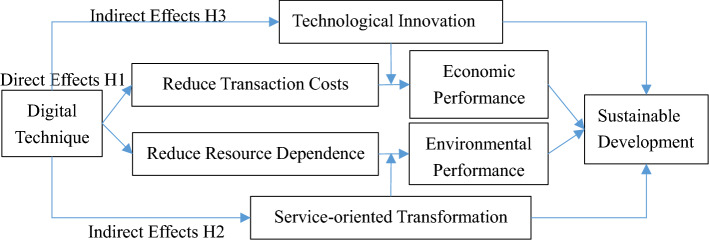
Theoretical basis for digital technology, service-oriented transformation and sustainable development.

The transaction cost theory and resource dependence theory are the theoretical foundations for analyzing the impact of digital technology on sustainable development. This article will elaborate on the mechanism of digital technology's impact on service-oriented transformation and sustainable development from both direct and indirect effects (as shown in Fig. 1).

Firstly, digital technology can help alleviate information asymmetry and reduce transaction costs. According to the transaction cost theory, in the case of information asymmetry, both parties may face higher transaction costs, which to some extent hinders production and business activities^40^. There are a lot of transaction costs in market transactions, which make it difficult to achieve some transactions, restrict the production and business activities of enterprises, and affect the business performance of enterprises. Relying on abundant data resources and information, digital technology can reduce information asymmetry and significantly reduce the search, information, negotiation, and supervision costs of transactions between supply and demand parties, thereby reducing the transaction costs of enterprises. The reduction of transaction costs improves enterprise productivity and also helps enterprises achieve economies of scale and scope. On one hand, the Internet of Things widely connects equipment, terminals, upstream and downstream of the industrial chain and consumers to improve the efficiency of data and information transmission. Big data analysis can optimize resource allocation and production processes for enterprises to improve production efficiency. The platform economy based on digital technology establishes a direct connection between enterprises and consumers, helps enterprises expand the scale of users, achieves the goal of "small profits but quick turnover", and thus realizes economies of scale. On the other hand, the digital economy can break the limitations of related products. Because of the digital technology reducing the information asymmetry between enterprises and consumers, enterprises can not only provide consumers with a large number of products of a single variety, but also provide a small number of products and services of multiple varieties for small demand, greatly reducing the production and sales costs of enterprises, so as to achieve economies of scope. It can be seen that digital technology plays an important role in reducing transaction costs, promoting economies of scale and scope, and thereby improving the economic performance of enterprises.

Secondly, digital technology can improve resource utilization efficiency and reduce dependence on the environment. The resource dependence theory suggests that organizations cannot achieve self-awareness and self-sufficiency, and must interact and exchange material, information, energy, and other aspects with organizers who control resources, thereby forcing organizations to become dependent on the external environment^41,42^. The application of digital technology enables the monitoring of the entire production process, strengthens key control, and reduces the dependence of enterprises on environmental resources. Traditional environmental governance policies and methods often focus on end-of-life governance, such as increasing environmental investment and increasing decontamination equipment, making it difficult to achieve process control of environmental governance. The application of artificial intelligence, the Internet, and other technologies in the manufacturing industry can not only reduce product development time and costs, but also achieve process control and lifecycle management^43^, helping manufacturing enterprises fully utilize circular resources, including circular procurement, circular design, recycling, and remanufacturing, thereby achieving a circular economy^44,45^ and improving environmental performance of enterprises.

#### H1

Digital technology helps improve economic and environmental performance of enterprises, and promotes sustainable development of enterprises.

### Digital technology, servitization, and environmental performance

According to the theory of resource dependence, digital technology can also reduce dependence on environmental resources by increasing the supply of services to manufacturing enterprises. New generation digital technologies such as big data, the Internet of Things, and artificial intelligence have realized the interconnection of everything, which not only improves the production efficiency, but also gathers massive data from all nodes and links of the manufacturing value chain, supply chain, and business ecology, becoming an important asset of enterprises^46^, helping to promote the service-oriented transformation of manufacturing enterprises. In terms of production, digital technology can increase the intelligent and flexible design of products to meet the diverse and customized product needs of consumers, and reduce the manufacturing cost and selling price of products. In terms of services, intelligent backend production, customer service, and software systems can provide users with diverse and continuously upgraded services, such as personalized customization, system solutions, product performance maintenance and optimization, greatly improving the variety and efficiency of manufacturing enterprise services.

Digital technology has extensive application value in enterprise production and management. Research has found that it has advantages that traditional production models cannot compare in improving production efficiency, increasing service supply and service targets. The Internet of Things, software, and other technologies play an important value creation role in intelligent service solutions, optimization, and control of production processes^47,48^, which can monitor the operational status and fault warnings of products and equipment in real-time, and provide customers with more after-sales services such as product maintenance. At the same time, the application of digital technology helps the manufacturing industry achieve new forms of innovation and business models, becoming the driving force for enterprise service-oriented development^38,49^. Some research found that digital technology has strong advantages in customer participation in the service process, providing customer service methods, and service delivery speed^50^. The service-oriented transformation of manufacturing enterprises not only enhances their competitive advantages, but also increases the recycling of products, improves resource and energy efficiency, and thus enhances the environmental performance of manufacturing enterprises. Based on the above analysis, propose the hypothesis:

#### H2

Digital technology promotes sustainable development of manufacturing enterprises by driving service-oriented transformation.

### Digital technology, technological innovation, and economic performance

According to Schumpeter's "creative destruction" theory, when the original economic state in a competitive environment is disrupted, the emergence of new organizational methods will lead to the destruction of the old organizational methods through competition. Intelligence and networking are the main characteristics of digital technology. The integration of digital technology and traditional industries have led to the emergence of new formats and models, which have impacted or even subverted traditional industries and formats, and are an important driving force for technological innovation. From a macro perspective, digital technology, under the "creative destruction" effect, optimizes industrial structure and drives the development of high-tech industries, and also stimulates the activity of mass entrepreneurship, becoming the main driving force for global technological innovation and economic development. From a micro perspective, the application of digital technology can achieve information integration and share in key links such as research and development, procurement, production, marketing, logistics, and services, alleviate information asymmetry between the consumer and innovative ends, facilitate precise docking of market demand and innovation resources, and enhance the technological innovation ability and efficiency of enterprises.

Specifically, the application of digital technology, on the one hand, affects the allocation, scale, and efficiency of enterprise factor resources by changing the status, role, and combination of different production factors. Driven by big data, enterprises increase more investment in R&D funds, R&D personnel and other innovative elements to obtain sustainable competitiveness and meet the diversified consumption needs of consumers. On the other hand, the application of digital technology drives product research and development transformation through channels such as reducing research and development costs, shortening research and development cycles, and consumer participation in product research and development, which further stimulates innovation in behavior insight, risk foresight, and business models, improving enterprise research and development efficiency. In addition, the application of digital technology provides an inclusive, collaborative and innovative platform for enterprise development, promotes information sharing, business cooperation and relationship coordination, and all economic entities can create value through big data coordination of resource organization and use process^51^, so as to improve the efficiency of collaborative innovation. Through sustainable innovation, manufacturing enterprises gain more competitive advantages and profit margins, thereby continuously improving economic performance and achieving sustainable development^52,53^.

#### H3

The application of digital technology promotes sustainable development of enterprises through technological innovation.

## Research and data methodology

### Econometric model

The sustainable development of enterprises is influenced not only by the application of digital technology and internal financial indicators, but also by factors such as external economic development and environmental policies. Referring to the related studies^25^, we utilize the data of listed companies in the Chinese manufacturing industry to empirically test the impact of digital technology application on corporate sustainable development. On the one hand, since the annual reports disclosed by listed companies are objective statements of the company's operation, the frequency of words related to digital technology involved in the annual reports of listed companies can be counted to reflect the degree of the company's digital technology application. On the other hand, Bloomberg, as three well-known ESG rating agencies, has covered nearly more than 1,400 Chinese listed companies, which provides reliable data support for the research of this paper. Based on this, this paper constructs the following econometric model:

1 $$ESG_{it} = \beta_{0} + \beta_{1} Digit_{it - 1} + \beta_{2} \sum {Controls}_{it} + \mu_{i} + \delta_{t} + \varepsilon_{it}$$

Among them, $i$ represents the enterprise, $t$ represents the year, $\mu_{i}$ and $\delta_{t}$ represents the fixed effect of the enterprise and the fixed effect of the year, respectively. $ESG_{it}$ represents a sustainable development indicator for enterprises, measured by the Bloomberg ESG rating index. $Digit_{it - 1}$ represents the indicator of digital technology application. In order to mitigate the interference of endogeneity issues on estimation results, this indicator is subjected to a lag of one period. $Controls_{it}$ represents relevant control variables, including corporate financial indicators, economic development, environmental policies, etc.

Theoretical analysis has found that the application of digital technology mainly affects sustainable development through channels such as technological service-oriented transformation and innovation. On the one hand, the application of digital technology can provide customers with services such as technical support, maintenance, renovation and scrapping, which will help to improve Resource efficiency, prolong life and improve product recycling^54^, thus affecting the sustainable development of enterprises. On the other hand, enterprises use digital technologies such as the Internet of Things and big data to improve product design, monitor and track product activities, which plays an important role in reducing research and development costs, shortening research and development cycles, and consumers' participation in product research and development, thus improving the competitiveness and innovation efficiency of organizations. In order to test the impact mechanism of enterprise digital technology application on enterprise sustainable development, on the basis of model (1), the following mesomeric effect model is constructed:

2 $$M_{it} = \alpha_{0} + \alpha_{1} Digit_{it - 1} + \alpha_{2} \sum {Controls_{it} } + \mu_{i} + \delta_{t} + \varepsilon_{it}$$

3 $$ESG_{it} = \gamma_{0} + \gamma_{1} Digit_{it - 1} + \gamma_{2} M_{it} + \gamma_{3} \sum {Controls_{it} } + \mu_{i} + \delta_{t} + \varepsilon_{it}$$

Among them, $M_{it}$ is an intermediary variable, mainly including the servitization and technological innovation indicators, while other variables are consistent with model (1). If the estimated coefficient of the core explanatory variable $\gamma_{1}$ is still significant after adding the intermediary variable, then there is a mesomeric effect. For $\beta_{1}$ and $\gamma_{1}$, if one of them is not significant, a secondary test using the Bootstrap method is required. If the 95% confidence interval obtained when testing the mesomeric effect does not contain 0, it indicates that the mesomeric effect is significant. On the contrary, there is no mesomeric effect.

### Variable design

#### Sustainable development

Most existing literature reflects corporate sustainability from three aspects: economy, environment, and social responsibility^55,56^. The ESG rating index is an effective indicator for third-party organizations to comprehensively evaluate companies from three aspects: environmental performance, social responsibility, and corporate governance. Referring to correlational research^57^, the Bloomberg ESG score was used to reflect the sustainable development of enterprises. On the one hand, Bloomberg is a globally renowned financial information service provider, and as a third-party institution, its ESG evaluation of Chinese enterprises is relatively objective and widely used. On the other hand, the rating index collects company information through public channels such as company annual reports, sustainable development reports, and company official websites, forming three sub indicators of environment, society, and corporate governance, providing data protection for studying the impact of various aspects of sustainable development of enterprises. The higher the Bloomberg ESG score, the better the company's ESG performance and sustainable development performance. The Bloomberg ESG rating also reports three sub scores: corporate environmental performance, social responsibility, and corporate governance, which are used to measure the company's environmental performance, social performance, and economic performance.

#### Application of digital technology

This article uses the frequency of keywords related to the application of digital technology to measure the degree of digital technology application (*Digit*). The application of enterprise digital technology is difficult to directly quantify, and some studies use text information from company annual reports for approximate estimation^58^. This is because the annual reports disclosed by listed companies are statements based on actual operating conditions. The more content related to the application of digital technology is involved in the company's business process that year, the more relevant keywords will be involved in the annual report. Therefore, by counting the frequency of keywords related to the application of digital technology, the company's digital technology application situation can be objectively reflected. Based on this method, this article establishes digital technology application keywords (Table 1) by referencing existing literature, important policy documents, research reports, etc., and uses Python crawler function to collect keyword frequency in company annual reports. Due to the fact that some companies and years do not have relevant keywords, the data has a right skewness feature. Therefore, by adding 1 to take the logarithm, it constitutes a digital technology application indicator.

**Table 1:** Selection of key words for digital technology application.

Technical category	Keywords
Artificial intelligence technology	Artificial intelligence, business intelligence, image understanding, investment decision support system, intelligent data analysis, intelligent robot, machine learning, deep learning, language search, biometrics, face recognition, voice recognition, identity verification, automatic driving, natural language processing, intelligent wear, intelligent agriculture, intelligent transportation, intelligent medical care, intelligent customer service, smart home, intelligent investment consultant, intelligent cultural tourism, intelligent environmental protection smart grid, smart marketing, smart energy
Big data technology	Big data, data mining, text mining, data and information visualization, heterogeneous data, credit reporting, augmented reality, mixed reality, virtual reality
Cloud computing technology	Cloud computing, stream computing, graph computing, memory computing, multi-party security computing, brain like computing, green computing, cognitive computing, fusion architecture, 100 million level concurrency, EB level storage, internet of things, information physical system
Blockchain technology	Blockchain, digital currency, distributed computing, differential privacy technology, smart financial contract, internet finance, digital finance, fintech, financial technology, quantitative finance, open banking
Internet technology	Mobile internet, industrial internet, mobile internet, internet healthcare, E-Commerce, mobile payment, third-party payment, NFC payment, B2B, B2C, C2B, C2C, O2O, internet connectivity, digital marketing, unmanned retail

#### Control variables

The control variables include corporate financial indicators, regional economic development levels, and environmental policies. Firstly, from the perspective of internal environment, sustainable development of enterprises is closely related to corporate profitability, market value, corporate financial, etc. Therefore, referring to existing research^59^, the financial indicators selected in this article include enterprise size (*Size*), return on total assets (*ROA*), asset liability ratio (*ALR*), top shareholder ratio (*Top1*), proportion of intangible assets (*Itang*), investment expenditure ratio (*Invt*), Tobinq, age, and duality. Secondly, from the perspective of external environment, regional economic development and environmental policies are important factors that affect the sustainable development of enterprises. Therefore, the indicators of regional economic development in this article include per capita GDP (*RGDP*) and industrial structure (*Struc*). The environmental policy indicators are measured by the intensity of regional environmental regulations (*EP*), and the specific algorithm is to measure the proportion of investment completed in industrial pollution control to industrial added value. The definitions of core variables and control variables are shown in Table 2.

**Table 2:** Definition of core and control variables.

Variable name	Symbolic	Variable definition
Sustainable development	*ESG*	Bloomberg ESG score logarithmic
Application of digital technology	*Digit*	The logarithm of the total frequency of digital technology application keywords in the company's annual report plus 1
Enterprise size	*Size*	The logarithm of the total asset size of the enterprise
Total return on assets	*ROA*	Net profit/total assets
Asset liability ratio	*ALR*	Liabilities/total assets
Proportion of the largest shareholder	*Top1*	The proportion of the largest shareholder to the total shares
Proportion of intangible assets	*Itang*	Net intangible assets/total assets
Investment expenditure rate	*Invt*	Cash paid for the purchase of fixed assets, intangible assets, and other long-term assets /total assets
Tobin q	*Tobin*	Sum of total market value and total liabilities of enterprises / total assets
Enterprise age	*Age*	The logarithm of the years of enterprise existence
Duality	*Duality*	Whether to concurrently serve as Chairman and General Manager
Per capita GDP	*RGDP*	Log of regional per capita GDP
Industrial structure	*Struc*	Proportion of Secondary sector of the economy in GDP
Environmental policy	*EP*	Investment completed in industrial pollution control / Output of industrial *100%

### Data sources and descriptive statistics

This article selects China A-share manufacturing listed companies from 2011 to 2020 as the initial research sample. Considering that the manufacturing industry is an important lifeline of a country's economic development and the main battlefield of technological innovation, and the scope of this article's perspective involves the fields of enterprise digitization and service-oriented, this article selects the manufacturing industry as the research sample. The financial characteristics related indicators data of listed companies in this article are sourced from the CSMAR database, while indicators such as regional economic development and environmental policies are mainly sourced from the statistical yearbooks of various provinces and cities in China and the website of the National Bureau of Statistics. We processed the initial samples as follows: (1) We excluded samples with ST, * ST, and PT treatments during the operating period; (2) Excluding samples of IPO listings and delisting in the past two years; (3) Delete samples with severe missing variables and asset liability ratio greater than 1. Through the above processing, this article ultimately obtained a total of 5314 samples from 607 listed companies. In order to avoid the interference of extreme, all continuous variables are shrunk by 1%. Table 3 reports the descriptive statistical results of the main variables.

**Table 3:** The descriptive statistical results of the main variables.

Variable	Obs	Mean	SD	Median	Min	Max
*ESG*	5314	2.995	0.313	3.008	2.207	3.798
*Digit*	5314	0.762	0.992	0	0	3.871
*Size*	5314	22.89	1.203	22.78	19.86	25.52
*ROA*	5314	0.0490	0.0600	0.0410	−0.201	0.209
*ALR*	5314	0.440	0.191	0.446	0.0520	0.896
*Top1*	5314	0.361	0.151	0.350	0.0900	0.732
*Itang*	5314	0.0440	0.0320	0.0370	0.00100	0.197
*Invt*	5314	0.0510	0.0430	0.0390	0.00200	0.223
*Tobin*	5314	2.043	1.301	1.602	0.886	8.126
*Age*	5314	2.825	0.352	2.890	1.386	3.466
*Duality*	5314	0.237	0.425	0	0	1
*RGDP*	5314	11.08	0.451	11.07	10.09	12.01
*Struc*	5314	0.406	0.0940	0.431	0.158	0.536
*EP*	5314	0.208	0.157	0.159	0.00900	0.827

## Analysis and discussion

### Benchmark regression results

Table 4 reports the estimated results of the impact of digital technology applications on sustainable development. This article adopts a progressive regression strategy. Column (1) only controls for firm and year fixed effects, column (2) adds relevant control variables, and column (3) adds regional fixed effects. The results show that the estimation coefficients of digital technology application on sustainable development are positive and significant at the 5% level. This means that the higher the degree of application of digital technology, the better the sustainable development performance of enterprises, and there is a significant positive correlation between the two. Columns (4)—(6) respectively report the impact of digital technology application on three sub categories: environmental performance, social performance, and economic performance. It can be seen that digital technology application mainly improves environmental performance and economic performance, but has no significant impact on social performance. From this, it can be seen that the application of digital technology has increased enterprise research and development, after-sales and other services, improved production and research and development efficiency, and thus improved environmental and economic performance of enterprises, verifying hypothesis H1.

**Table 4:** Benchmark regression results of the impact of digital technology application on sustainable development.

	(1)	(2)	(3)	(4)	(5)	(6)
	*ESG*	*ESG*	*ESG*	*ESG_E*	*ESG_S*	*ESG_G*
*L.Digit*	0.00951** (0.004)	0.00901**	0.00976**	0.0229**	0.00731	0.00396***
		(0.004)	(0.004)	(0.010)	(0.005)	(0.001)
*Size*		0.0830***	0.0802***	0.114***	0.118***	0.0144***
		(0.010)	(0.010)	(0.025)	(0.015)	(0.003)
*ROA*		0.0176	0.0205	0.0419	− 0.0421	0.0206
		(0.054)	(0.053)	(0.136)	(0.080)	(0.018)
*ALR*		− 0.127***	− 0.126***	− 0.168**	− 0.157***	− 0.0575***
		(0.033)	(0.033)	(0.083)	(0.048)	(0.012)
*Top1*		0.227***	0.233***	0.375***	0.231***	0.0816***
		(0.047)	(0.048)	(0.126)	(0.070)	(0.020)
*Itang*		− 0.0713	− 0.136	0.245	− 0.279	− 0.147**
		(0.171)	(0.169)	(0.421)	(0.255)	(0.057)
*Invt*		− 0.180**	− 0.162**	− 0.539**	− 0.123	0.0169
		(0.083)	(0.080)	(0.210)	(0.119)	(0.031)
*Tobin*		0.0107***	0.0111***	0.0199**	0.0126**	− 0.00110
		(0.003)	(0.003)	(0.010)	(0.005)	(0.001)
*Age*		0.0129	0.0175	− 0.132	0.0385	− 0.0806***
		(0.051)	(0.051)	(0.144)	(0.076)	(0.017)
*Duality*		− 0.00722	− 0.00768	− 0.0223	− 0.00860	− 0.00307
		(0.008)	(0.008)	(0.023)	(0.012)	(0.003)
*RGDP*		0.0331	0.0420	− 0.280	− 0.0310	− 0.0192
		(0.033)	(0.073)	(0.188)	(0.107)	(0.029)
*Struc*		0.0139	− 0.265	0.354	− 0.187	− 0.0180
		(0.126)	(0.214)	(0.587)	(0.309)	(0.082)
*EP*		0.0540**	0.0510**	0.0709	0.0789**	0.0112
		(0.023)	(0.023)	(0.058)	(0.036)	(0.009)
*_cons*	3.013***	0.653	0.712	2.862	0.663	3.905***
	(0.003)	(0.448)	(0.836)	(2.118)	(1.203)	(0.327)
Firm FE	Yes	Yes	Yes	Yes	Yes	Yes
Year FE	Yes	Yes	Yes	Yes	Yes	Yes
Province FE	No	No	Yes	Yes	Yes	Yes
Adj. R^2^	0.781	0.789	0.790	0.746	0.744	0.807
N	4700	4700	4700	4260	4676	4700

(1) ***, **, *respectively represent significant levels at 1%, 5%, and 10%; (2) The brackets below the coefficients indicate the standard error of clustering robustness; (3) L. Digit represents a lag period of digital technology application indicators.

In terms of controlling variables, financial indicators such as enterprise size, top shareholder shareholding ratio, and enterprise value have a significant positive impact on sustainable development. This indicates that sustainable development of enterprises is closely related to enterprise size and operating conditions. Meanwhile, environmental policies (*EP*) have a significant positive impact on the sustainable development of enterprises, indicating that the stricter external environmental regulations, the more actively enterprises improve environmental performance to meet environmental regulatory requirements, and the better their sustainable development performance. In addition, the increase in asset liability ratio (*ALR*) is not conducive to the sustainable development of enterprises, which means that the increase in debt may threaten the long-term development of enterprises.

### Robustness testing

To test the robustness of benchmark regression results, methods such as replacing variables, adjusting samples, and controlling for external shocks were used for estimation.

1. Replace variables. For the sustainable development, the Huazheng ESG Rating Index (*ESG_H*) is used as a substitute variable for robustness testing. The specific approach is to divide the Huazheng ESG rating index into nine levels, AAA-C, based on their advantages and disadvantages. They are ranked from high to low and assigned values ranging from 9 to 1, meaning that the higher the enterprise's rating level, the greater the assigned value. For digital technology application indicators, the ratio of the frequency of digital technology application keywords to the total number of texts in the annual report (*Digit_r*) is used as a substitute variable. In Table 5, columns (1) and (2) report the estimated results after replacing the dependent variable and digital technology application indicators, indicating that the impact coefficient of digital technology application on sustainable development is still significantly positive.
2. Adjust the sample. In all manufacturing industries, computer, communication, and other electronic equipment manufacturing are closely related to digital technology. Therefore, in order to eliminate this factor that may have an impact on the regression results, this industry was excluded from the sample. The estimated results of column (3) in Table 5 show that after excluding computer-related manufacturing, the impact of digital technology application on sustainable development is still significantly positive.
3. Control external shocks. During the sample period, there were significant fluctuations in the Chinese stock market from 2014 to 2015. This external impact may affect the investment and financing environment of enterprises, as well as their business decisions, thereby affecting their sustainable development. Therefore, to eliminate the impact of external shocks, the samples from 2014 and 2015 were deleted. The results of column (4) in Table 5 show that after excluding external impact factors, the impact of digital technology application on sustainable development is still significantly positive.

**Table 5:** Estimation results of robustness test.

	(1)	(2)	(3)	(4)
	*ESG_H*	*ESG*	*ESG*	*ESG*
*L.Digit*	0.0466**		0.00959**	0.00996**
	(0.019)		(0.004)	(0.005)
*Digit_r*		0.112*		
		(0.058)		
*Size*	0.335***	0.0840***	0.0818***	0.0797***
	(0.041)	(0.009)	(0.011)	(0.011)
*ROA*	0.203	− 0.0133	0.0374	0.0499
	(0.318)	(0.057)	(0.059)	(0.062)
*ALR*	− 1.052***	− 0.125***	− 0.139***	− 0.133***
	(0.157)	(0.034)	(0.035)	(0.037)
*Top1*	0.373	0.175***	0.254***	0.212***
	(0.248)	(0.045)	(0.053)	(0.057)
*Itang*	− 0.876	− 0.0574	− 0.124	− 0.285
	(0.683)	(0.165)	(0.186)	(0.207)
*Invt*	1.446***	− 0.0650	− 0.0883	− 0.167*
	(0.382)	(0.082)	(0.090)	(0.093)
*Tobin*	0.0429***	0.0142***	0.0106***	0.00662
	(0.014)	(0.003)	(0.004)	(0.004)
*Age*	− 0.426**	0.0409	0.00713	0.0479
	(0.200)	(0.050)	(0.058)	(0.057)
*Duality*	0.0780*	− 0.00762	− 0.00702	− 0.00151
	(0.041)	(0.008)	(0.009)	(0.009)
*RGDP*	0.134	− 0.0429	0.0934	0.0327
	(0.345)	(0.078)	(0.077)	(0.088)
*Struc*	− 2.615***	− 0.749***	− 0.264	− 0.292
	(0.954)	(0.218)	(0.224)	(0.256)
*EP*	− 0.148	0.0412*	0.0471*	0.0567**
	(0.115)	(0.024)	(0.025)	(0.028)
*_cons*	− 2.328	1.695*	0.138	0.782
	(3.825)	(0.883)	(0.892)	(1.008)
Firm FE	Yes	Yes	Yes	Yes
Year FE	Yes	Yes	Yes	Yes
Province FE	Yes	Yes	Yes	Yes
Adj. R^2^	0.495	0.754	0.786	0.799
N	5306	5314	4084	3768

(1) ***, **, * respectively represent significant levels at 1%, 5%, and 10%; (2) The brackets below the coefficients indicate the standard error of clustering robustness; (3) L. Digit represents a lag period of digital technology application indicators.

### Endogeneity discussion

In the benchmark regression model, lagging the core explanatory variable digital technology application indicator for one period can alleviate endogeneity bias caused by reverse causality to some extent, but it may still lead to endogeneity problems due to unobservable missing variables. For this reason, this paper draws on the research ideas of correlational research^27,60^, and uses the "Broadband China" policy as a quasi Natural experiment to test the impact of digitalization on the sustainable development of enterprises. The Ministry of Industry and Information Technology of China and the National Development and Reform Commission jointly issued the "Management Measures for Creating" Broadband China "Demonstration Cities (Urban Agglomerates)" (hereinafter referred to as the "Measures"). Based on indicators such as household broadband access capacity, broadband penetration rate, mobile phone penetration rate, and broadband user penetration rate, 120 "Broadband China" demonstration cities (clusters) were selected in three batches in 2014, 2015, and 2016. The "Measures" clearly pointed out that the key points of the construction of demonstration cities include improving the speed and application level of broadband networks, promoting the continuous improvement of the broadband network industry chain, enhancing the security guarantee ability of broadband networks, etc. It is a typical constructive pilot city, which puts forward higher requirements for the development and construction of the digital economy of pilot cities. The policy can be seen as a quasi Natural experiment. Therefore, this article constructs the following model to test the impact of digitalization on the sustainable development of enterprises:

4 $$ESG_{ict} = \gamma_{0} + \gamma_{1} Digitcity_{ic} *Post_{t} + \gamma_{2} \sum {Control_{ict} } + \mu_{i} + \delta_{t} + \varepsilon_{it}$$

Among them, $i$ represents the enterprise, $c$ represents the city, $t$ represents the year, $\mu_{i}$ and $\delta_{t}$ represents the fixed effect of the enterprise and the fixed effect of the year, respectively. $Digitcity_{ic}$ indicates whether the city is a "Broadband China" demonstration city, if the city is determined to be a "Broadband China" demonstration city $Digitcity_{ic} = 1$, otherwise $Digitcity_{ic} = 0$; $Post_{t}$ indicates the year of being designated as a "Broadband China" demonstration city, determined as the year of the demonstration city and subsequent years $Post_{t} = 1$, otherwise $Post_{t} = 0$. The other indicators are consistent with model (1). Table 6 reports the estimated impact of the "Broadband China" policy on the sustainable development of enterprises. It can be seen that the broadband China demonstration city policy has a significant positive impact on the sustainable development of enterprises, indicating that digitization can help improve the sustainable development performance of enterprises.

**Table 6:** Estimated results of the sustainable development impact of enterprises based on "Broadband China".

	(1)	(2)
	*ESG*	*ESG*
Digitcity*Post	0.0249**	0.0231**
	(0.010)	(0.010)
*Size*		0.0843***
		(0.009)
*ROA*		− 0.0125
		(0.057)
*ALR*		− 0.124***
		(0.034)
*Top1*		0.177***
		(0.045)
*Itang*		− 0.0446
		(0.165)
*Invt*		− 0.0701
		(0.082)
*Tobin*		0.0141***
		(0.003)
*Age*		0.0388
		(0.050)
*Duality*		− 0.00835
		(0.008)
*RGDP*		− 0.0605
		(0.078)
*Struc*		− 0.725***
		(0.217)
*EP*		0.0389
		(0.024)
*_cons*	2.982***	1.871**
	(0.006)	(0.886)
Firm FE	Yes	Yes
Year FE	Yes	Yes
Province FE	Yes	Yes
Adj. R^2^	0.745	0.754
N	5314	5314

(1) ***, **, *respectively represent significant levels at 1%, 5%, and 10%; (2) The brackets below the coefficients indicate the standard error of clustering robustness.

**Figure 2:**
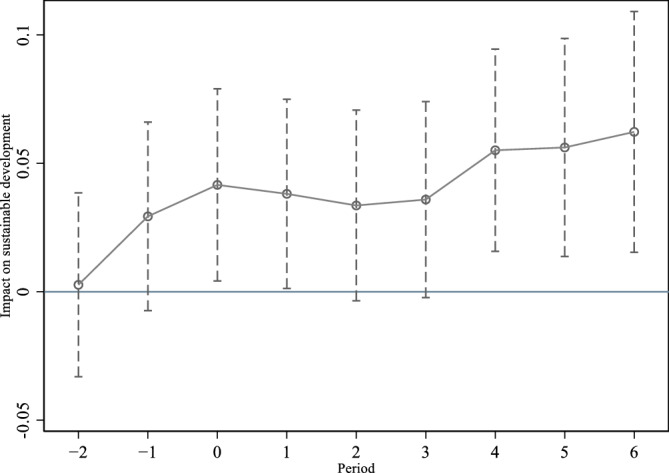
Parallel trend test chart.

When using the difference-in-difference model to test the impact of broadband China demonstration cities on sustainable development of enterprises, it is necessary to satisfy the parallel trend hypothesis, that is, before implementing the broadband China policy, the treatment group and the control group have a consistent trend of change. This paper uses the event study to test the parallel trend hypothesis. Figure 2 shows the parallel trend test. The vertical axis is the size of the estimation coefficient of the impact of broadband China's policy on the sustainable development of enterprises in different events, the horizontal axis is the relative time before and after the implementation of broadband China's policy, 0 represents the initial period of broadband China's policy implementation, and the dotted line above and below the hollow circle is the 90% confidence interval. The results in Fig. 1 show that before the implementation of the broadband China policy, the broadband China demonstration cities did not have a significant impact on the sustainable development of enterprises, and the estimated coefficient was around 0, indicating a parallel trend. After implementing the broadband China policy, the demonstration cities of broadband China have a significant positive impact on the sustainable development of enterprises, and this impact is even greater after the fourth phase.

## Further analysis

### Heterogeneity analysis

The impact of digital technology application on sustainable development may vary depending on the nature, scale, resource attributes, and industry agglomeration characteristics of enterprises. Therefore, this article further explores the impact of digital technology application on sustainable development under heterogeneous conditions.

1. Nature of the enterprise. There may be differences in the impact of digitalization on enterprises of different corporate nature. This article divides the sample into state-owned enterprises and private enterprises based on the actual controllers of the company. Columns (1) and (2) of Table 7 report the estimated heterogeneity of enterprise nature. Compared to state-owned enterprises, the application of digital technology in private enterprises has a greater impact on sustainable development and is significant at the 5% level. This means that private enterprises are more conducive to sustainable development through the application of digital technology. The reason for this is that private enterprises rely on digital technology to improve research and development efficiency, develop new channels, and provide more high-quality services, thereby enhancing their competitive advantage.
2. Enterprise scale. The heterogeneity of enterprise scale may also affect the digital technology application of enterprises. This article divides the sample into large enterprises and small and medium-sized enterprises based on their listing locations. According to the different sectors of the company's listing, companies listed on the SME board are classified as small and medium-sized enterprises (stocks with codes starting with 300 or 002), while the rest are classified as large enterprises. Columns (3) and (4) of Table 7 report the estimated heterogeneity of enterprise size. Compared to large enterprises, the application of digital technology in small and medium-sized enterprises has a more significant impact on sustainable development, which means that the application of digital technology in small and medium-sized enterprises performs better. Small and medium-sized enterprises face greater competitive pressure, and the application of digital technology plays an important role in reducing transaction costs and winning customer image, thereby enhancing the sustainable development of enterprises.
3. Resource attributes. The application of digital technology in different industries may also have different impacts. Environmental performance is a key component of sustainable development for enterprises, and resource-based industries are more dependent on the environment and may be more affected by the application of digital technology. This article divides the sample into resource based enterprises and non resource based enterprises based on industry attributes. Columns (5) and (6) of Table 7 report the estimation results of industry attribute heterogeneity, indicating that the application of digital technology has a significant positive impact on both resource-based and non-resource-based enterprises. However, the estimation coefficient of digital technology application in resource-based enterprises is larger, which means that the sustainable development of resource-based enterprises is more affected by digitization. Resource-based enterprises rely more on the environment. By using digital technology to improve production processes and achieve full monitoring of production processes, the environmental performance and sustainable development performance of resource-based enterprises have been greatly improved.

**Table 7:** Heterogeneity estimation results of the impact of digital technology application on sustainable development.

	(1)	(2)	(3)	(4)	(5)	(6)
	State owned	Private	Large	Small and medium-sized	Resource-based	Non- resource-based
*L.Digit*	0.00498	0.0130**	0.00174	0.0154**	0.0269**	0.00922**
	(0.006)	(0.005)	(0.005)	(0.006)	(0.012)	(0.004)
*Size*	0.0614***	0.112***	0.0820***	0.113***	0.0800***	0.0871***
	(0.014)	(0.015)	(0.013)	(0.016)	(0.021)	(0.011)
*ROA*	0.131	− 0.0924	0.0809	− 0.106	− 0.0246	0.0182
	(0.083)	(0.069)	(0.074)	(0.079)	(0.120)	(0.061)
*ALR*	− 0.0654	− 0.183***	− 0.0328	− 0.159***	− 0.119*	− 0.125***
	(0.052)	(0.045)	(0.044)	(0.052)	(0.069)	(0.037)
*Top1*	0.117*	0.350***	0.176***	0.263***	0.183**	0.239***
	(0.064)	(0.073)	(0.056)	(0.086)	(0.092)	(0.055)
*Itang*	− 0.0557	− 0.387*	− 0.0891	− 0.313	0.576	− 0.376**
	(0.247)	(0.225)	(0.198)	(0.258)	(0.453)	(0.175)
*Invt*	− 0.149	− 0.165*	− 0.207*	− 0.180	0.0455	− 0.212**
	(0.142)	(0.097)	(0.115)	(0.111)	(0.161)	(0.095)
*Tobin*	0.00906	0.0123***	0.00661	0.0138***	− 0.00589	0.0139***
	(0.006)	(0.004)	(0.005)	(0.005)	(0.010)	(0.004)
*Age*	0.136*	0.0180	0.119*	0.156*	− 0.138	0.0369
	(0.078)	(0.069)	(0.065)	(0.082)	(0.228)	(0.049)
*Duality*	− 0.0229*	0.00883	− 0.00431	− 0.0137	0.0294	− 0.0206**
	(0.014)	(0.010)	(0.011)	(0.012)	(0.018)	(0.009)
*RGDP*	− 0.139	0.351***	0.118	− 0.172	0.259*	− 0.0806
	(0.102)	(0.102)	(0.084)	(0.145)	(0.135)	(0.086)
*Struc*	− 0.0350	− 0.453	− 0.206	− 0.140	− 0.761	− 0.0166
	(0.279)	(0.357)	(0.242)	(0.464)	(0.477)	(0.233)
*EP*	0.0411	0.0750**	0.0571**	0.0373	0.0899**	0.0504*
	(0.032)	(0.036)	(0.027)	(0.046)	(0.043)	(0.027)
*_cons*	2.769**	− 3.410***	− 0.469	1.893	− 0.970	1.759*
	(1.153)	(1.208)	(1.006)	(1.627)	(1.734)	(0.967)
Firm FE	Yes	Yes	Yes	Yes	Yes	Yes
Year FE	Yes	Yes	Yes	Yes	Yes	Yes
Province FE	Yes	Yes	Yes	Yes	Yes	Yes
Adj. R^2^	0.789	0.782	0.788	0.768	0.777	0.794
N	2199	2490	2790	1910	957	3741

(1) ***, **, *Respectively represent significant levels at 1%, 5%, and 10%; (2) The brackets below the coefficients indicate the standard error of clustering robustness; (3) L. Digit represents the lag period of digital technology application indicators, and the dependent variable is the sustainable development indicator ESG.

(4) Industry agglomeration characteristics. There are significant differences in the clustering characteristics of resource elements used by different industries. The characteristics of labor-intensive industries are that they mainly rely on a large amount of labor in the production process and have a low dependence on technology and equipment. Capital-intensive industries require more capital investment. Technology-intensive industries are industries that develop with technological knowledge as the main production factor. Referring to some research^61^, the sample was divided into labor-intensive, capital-intensive, and technology-intensive industries. Table 8 reports the estimation results of heterogeneity in industry agglomeration characteristics, indicating that the application of digital technology has a significant positive impact on the sustainable development of capital-intensive industries, while the impact on labor-intensive and technology-intensive industries is not significant. The reason for this is that compared to labor-intensive industries, capital-intensive industries have capital advantages, thereby increasing the scope and impact of digital technology applications. However, due to the early stage of digital technology application, it has not yet had a significant impact on technology-intensive industries.

**Table 8:** Estimation results of heterogeneity of industry agglomeration characteristics.

	(1)	(2)	(3)
	Labor-intensive	Capital-intensive	Technology-intensive
*L.Digit*	0.0125	0.0192**	0.00813
	(0.009)	(0.009)	(0.005)
*Size*	0.109***	0.0906***	0.0841***
	(0.039)	(0.026)	(0.011)
*ROA*	0.0178	0.0614	− 0.0136
	(0.167)	(0.118)	(0.069)
*ALR*	− 0.359***	− 0.0584	− 0.120***
	(0.111)	(0.067)	(0.041)
*Top1*	0.621***	− 0.000950	0.223***
	(0.128)	(0.091)	(0.061)
*Itang*	− 0.557	− 0.350	− 0.0800
	(0.440)	(0.358)	(0.220)
*Invt*	− 0.104	− 0.0820	− 0.172*
	(0.238)	(0.166)	(0.101)
*Tobin*	0.0174**	0.0170	0.00836**
	(0.009)	(0.011)	(0.004)
*Age*	0.232*	− 0.288**	0.0380
	(0.138)	(0.116)	(0.064)
*Duality*	− 0.0352	0.0140	− 0.0118
	(0.024)	(0.020)	(0.010)
*RGDP*	− 0.0849	0.343**	− 0.0291
	(0.238)	(0.136)	(0.092)
*Struc*	0.342	− 0.792*	− 0.201
	(0.707)	(0.429)	(0.264)
*EP*	0.0873	0.0445	0.0584*
	(0.072)	(0.039)	(0.031)
*_cons*	0.478	− 1.633	1.334
	(2.745)	(1.609)	(1.061)
Firm FE	Yes	Yes	Yes
Year FE	Yes	Yes	Yes
Province FE	Yes	Yes	Yes
Adj. R^2^	0.765	0.773	0.796
N	598	1009	3054

(1) ***, **, *respectively represent significant levels at 1%, 5%, and 10%; (2) The brackets below the coefficients indicate the standard error of clustering robustness; (3) L. Digit represents the lag period of digital technology application indicators, and the dependent variable is the sustainable development indicator ESG.

### Mechanism analysis

Due to its powerful advantages in reducing transaction and information costs, and improving efficiency, digital technology has been widely applied in various aspects of enterprises, and has had a profound impact on research and development, production, operation, and management. Overall, the application of digital technology affects the sustainable development of enterprises through mechanisms such as service-oriented transformation and technological innovation. To test the mechanism of digital technology application, model (2) and model (3) mesomeric effect models are used for estimation.

Firstly, regarding the degree of servitization, this article is based on related studies^29,62^, and other studies to define and measure servitization, using the ratio of enterprise service revenue to main business revenue as a measure. The specific approach is to check the annual reports of manufacturing enterprises, including the name of the enterprise's operating products, product types, and business scope, to determine whether the enterprise is engaged in service business. Then, based on the composition of operating income, the main business income of the enterprise is divided into service income and non-service income, in order to calculate the degree of servitization indicators. Columns (1) and (2) of Table 9 report the estimated results of the impact of digital technology application on the sustainable development of enterprises through service-oriented transformation. It can be seen that digital technology application has significantly increased the service income of enterprises and promoted their service-oriented transformation. At the same time, service-oriented transformation has a significant role in promoting the sustainable development of enterprises. Manufacturing enterprises use digital technology to improve production processes, provide more service support for customers, improve enterprise environmental performance by improving resource efficiency, increasing product recycling, etc. Thus improve enterprise sustainable development performance, and verifying the hypothesis H2.

**Table 9:** Mechanism analysis of the impact of digital technology application on sustainable development.

	(1)	(2)	(3)	(4)
	*Servitization*	*ESG*	*Innovation*	*ESG*
*L.Digit*	0.00318***	0.00817**	0.0521***	0.00929**
	(0.001)	(0.004)	(0.019)	(0.004)
*Servitization*		0.0903*		
		(0.052)		
*Innovation*				0.00889***
				(0.003)
*Size*	− 0.00537	0.0864***	0.555***	0.0753***
	(0.005)	(0.009)	(0.050)	(0.010)
*ROA*	− 0.0169	− 0.00641	− 0.942***	0.0288
	(0.015)	(0.057)	(0.270)	(0.053)
*ALR*	0.0157	− 0.127***	− 0.364**	− 0.123***
	(0.016)	(0.034)	(0.170)	(0.033)
*Top1*	− 0.0627**	0.165***	0.253	0.231***
	(0.032)	(0.044)	(0.252)	(0.048)
*Itang*	− 0.0943	0.000595	2.582***	− 0.159
	(0.061)	(0.165)	(0.680)	(0.169)
*Invt*	− 0.00728	− 0.0755	0.0383	− 0.162**
	(0.026)	(0.084)	(0.404)	(0.080)
*Tobin*	− 0.000214	0.0136***	0.00435	0.0110***
	(0.001)	(0.003)	(0.015)	(0.003)
*Age*	0.0677***	0.0334	− 0.751***	0.0242
	(0.020)	(0.050)	(0.219)	(0.052)
*Duality*	− 0.00155	− 0.00711	− 0.0722*	− 0.00704
	(0.002)	(0.008)	(0.042)	(0.008)
*RGDP*	− 0.0373**	− 0.0351	− 0.414	0.0457
	(0.019)	(0.032)	(0.384)	(0.073)
*Struc*	0.0387	− 0.430***	− 0.512	− 0.260
	(0.056)	(0.126)	(1.212)	(0.213)
*EP*	0.000558	0.0371	0.0965	0.0502**
	(0.005)	(0.024)	(0.116)	(0.023)
*_cons*	0.362	1.446***	− 3.884	0.747
	(0.267)	(0.445)	(4.341)	(0.836)
Firm FE	Yes	Yes	Yes	Yes
Year FE	Yes	Yes	Yes	Yes
Province FE	Yes	Yes	Yes	Yes
Adj. R^2^	0.707	0.752	0.806	0.791
N	4700	4700	4700	4700

(1) ***, **, *respectively represent significant levels at 1%, 5%, and 10%; (2) The brackets below the coefficients indicate the standard error of clustering robustness; (3) L. Digit represents a lag period of digital technology application indicators.

Secondly, for technological innovation, the number of enterprise invention patent applications is measured by referring to existing literature. The reason for this approach is that, on the one hand, according to the essence of innovation, the number of patents owned by enterprises better reflects their innovation capabilities. However, the time from patent application to authorization varies from a few months to several years, so there is a certain lag in patent acquisition. The number of patent applications can be used as a substitute variable for enterprise technological innovation. On the other hand, compared to utility patents and design patents, invention patents require higher requirements and have a greater impact on the sustainable development of enterprises. Therefore, selecting the number of invention patent applications as an indicator of enterprise technological innovation has obvious advantages. Columns (3) and (4) of Table 9 report the estimated results of the impact of digital technology applications on the sustainable development of enterprises through technological innovation. It can be seen that digital technology applications significantly improve the technological innovation of enterprises, while technological innovation has a significantly positive impact on the sustainable development of enterprises. The Mesomeric effect model test shows that there is a mechanism for digital technology applications to affect the sustainable development of enterprises through technological innovation, and verifies the hypothesis H3.

## Discussions

The sustainable development of enterprises is to create economic value in terms of increasing added value or reducing costs, and at the same time to extend the life cycle of products in terms of reduction, reuse and recycling^63^, so as to reduce the negative impacts of environmental pollutions and emissions. According to transaction cost theory and resource dependence theory, digital technology improves the economic performance of enterprises by reducing transaction costs, and at the same time, it can also improve the efficiency of resource utilization and reduce the waste of resources, so as to improve the environmental performance of enterprises. The findings of this paper are basically consistent with those who found that digital technology improves resource efficiency and reduces resource waste in the process of product production and use^64,65^. In addition to examining the environmental performance resulting from the application of digital technology, this paper further analyzes the economic performance that may result from it.

Further, the paper also explores the key mechanisms for enhancing corporate sustainability from the interaction between digital technology applications and servitization, providing new strategic directions for companies to cope with increasingly stringent environmental regulations. On the one hand, servitization can reduce the amount of waste and raw material consumption during the product life cycle, and improve the environmental performance of enterprises by extending the product life and increasing the product recycling service. On the other hand, digital technology application can better help the transformation of enterprises' servitization, which is more advantageous for realizing the circular economy and sustainable development. Currently, research in this area focuses on the positive environmental impacts of servitization in manufacturing companies^14,36,37^, and the research in this paper is a useful addition to this area. Promoting the application of digital technology in enterprises and guiding the transformation of manufacturing enterprises into service-oriented enterprises has become an important direction for the sustainable development of countries, especially developing countries, in the face of economic globalization and worsening global environmental problems.

Finally, the empirical study in this paper finds that digital technology application has a greater impact on the sustainable development of resource-based enterprises and capital-intensive industries. Some research also confirms that the environmental performance of high-tech resource-based enterprises is more prominently affected by digital transformation^66^. Resource-based enterprises are highly dependent on the environment, and green transformation is difficult. The conclusions of this paper can provide a basis for relevant government departments to formulate policies to optimize industrial structure and promote green transformation of resource-based enterprises.

## Conclusion and research prospects

The application scenarios of digital technology are becoming increasingly widespread. While improving enterprise production efficiency, innovation efficiency, and operational efficiency, it also has characteristics such as economies of scale, economies of scope, and reducing transaction costs, which have a significant impact on the sustainable development of enterprises. Based on transaction cost theory and resource dependence theory, this paper explores the impact of digital technology application on servitization transformation and sustainable development, and empirically analyzes it with the data of listed manufacturing enterprises in China from 2011 to 2020. The results showed that, firstly, the application of digital technology has promoted sustainable development of enterprises, mainly manifested as a significant impact on their economic and environmental performance, which is more significant in small and medium-sized private enterprises. Secondly, resource-based enterprises are more dependent on the environment and therefore more affected by digital technology. Thirdly, at present, the sustainable development of capital intensive manufacturing is more affected by digital technology, and the impact of technology intensive enterprises has not yet been apparent. Fourthly, from the perspective of its mechanism of action, the application of digital technology has become the driving force and promoter of service-oriented transformation, helping enterprises propose intelligent service solutions, producting maintenance and recycling for customers, thereby driving service-oriented transformation and influencing the sustainable development of enterprises through technological innovation.

The research in this paper can provide useful insights for the strategy of sustainable development of enterprises. Firstly, adhering to the digital transformation strategy and improves the enterprise's ability to apply digital technology, which in turn generates new business growth points and competitive advantages. This paper finds that the application of digital technology promotes the sustainable development of enterprises by reducing transaction costs and improving the efficiency of resource utilization, and at the same time, the application of digital technology also generates new service businesses and improves the efficiency of traditional services, thus accelerating the transformation of enterprise services. Therefore, enterprises should increase digital technology investment and management. Secondly, for state-owned enterprises with scale advantages, they should play a leading role in the application of digital technology to drive the upstream and downstream digital transformation of the industrial chain. For small and medium-sized private enterprises, they should actively explore the road of digital transformation, and with the help of internal and external resources as well as policy support, they should continuously improve the ability of applying digital technology and the level of service, and realize sustainable development by transforming the traditional mode of production. Most SMEs have little experience in digital transformation, have a low success rate, and lack the necessary resources, skills and assessment of the value of digital technology application, SMEs have greater difficulties in adopting new technologies^67^. Therefore, the government should give more financial and fiscal support to the digital transformation of SMEs to create new growth drivers for promoting green transformation and sustainable development.

This paper explores the path of enterprise sustainable development from the perspective of the interaction between digital technology and servitization, and the next research can further consider the study of enterprise sustainable development strategies from the perspectives of supply chain efficiency, innovation efficiency, etc. And the discussion on how to improve the ability of digital technology and resolve the digital paradox will be of great benefit to the enterprise's digitalization strategy. Considering the availability of data and the urgency of enterprise transformation in the new strategic context (Dual-carbon target), this paper adopts the data of Chinese manufacturing enterprises for analysis, which has some limitations. The next study could be based on a comparative analysis of digital technology adoption and sustainable development of enterprises in different countries or industries, in order to make more targeted practical recommendations.

## Data Availability

The datasets used and/or analysed during the current study available from the corresponding author on reasonable request.
